# Attention-Based Lightweight YOLOv8 Underwater Target Recognition Algorithm

**DOI:** 10.3390/s24237640

**Published:** 2024-11-29

**Authors:** Shun Cheng, Zhiqian Wang, Shaojin Liu, Yan Han, Pengtao Sun, Jianrong Li

**Affiliations:** 1 Changchun Institute of Optics, Fine Mechanics and Physics, Chinese Academy of Sciences, Relative Pose Precision Measurement Laboratory, Jilin 130033, China; chengshun22@mails.ucas.ac.cn (S.C.); wangzhiqian@ciomp.ac.cn (Z.W.); liusj@ciomp.ac.cn (S.L.); hanyan23@mails.ucas.ac.cn (Y.H.); sunpengtao23@mails.ucas.ac.cn (P.S.); 2 Graduate School, University of Chinese Academy of Sciences, Beijing 100049, China

**Keywords:** underwater target recognition, lightweight model, PSA, YOLOv8

## Abstract

Underwater object detection is highly complex and requires a high speed and accuracy. In this paper, an underwater target detection model based on YOLOv8 (SPSM-YOLOv8) is proposed. It solves the problems of high computational complexities, slow detection speeds and low accuracies. Firstly, the SPDConv module is utilized in the backbone network to replace the standard convolutional module for feature extraction. This enhances computational efficiency and reduces redundant computations. Secondly, the PSA (Polarized Self-Attention) mechanism is added to filter and enhance the polarization of features in the channel and spatial dimensions to improve the accuracy of pixel-level prediction. The SCDown (spatial–channel decoupled downsampling) downsampling mechanism is then introduced to reduce the computational cost by decoupling the space and channel operations while retaining the information in the downsampling process. Finally, MPDIoU (Minimum Point Distance-based IoU) is used to replace the CIoU (Complete-IOU) loss function to accelerate the convergence speed of the bounding box and improve the bounding box regression accuracy. The experimental results show that compared with the YOLOv8n baseline model, the SPSM-YOLOv8 (SPDConv-PSA-SCDown-MPDIoU-YOLOv8) detection accuracy reaches 87.3% on the ROUD dataset and 76.4% on the UPRC2020 dataset, and the number of parameters and amount of computation decrease by 4.3% and 4.9%, respectively. The detection frame rate reaches 189 frames per second on the ROUD dataset, thus meeting the high accuracy requirements for underwater object detection algorithms and facilitating lightweight and fast edge deployment.

## 1. Introduction

The ocean is regarded as a treasure trove of resources and a strategic space to support the future development of the country, and it is becoming increasingly important to explore marine resource development technology. Underwater optical image target detection technology has been widely used in marine resource exploration, scientific marine research, underwater robot navigation, marine environmental monitoring, and other fields [1]. Compared with sonar technology, underwater optical imaging technology offers a higher image resolution, rich color and texture information, and good real-time performance, and compared with electromagnetic induction technology, it has a wide range of applications and is not limited to specific materials. Underwater optical target recognition systems are generally installed on underwater robots and embedded systems [2], but the underwater working environment is harsh, and the computing resources and power supply are limited. It is therefore very important to reduce the weight of the model. In addition, since the target moves in the water, and the speed of this movement is affected by factors such as buoyancy, the shape of the target, currents, and large fish, when the target appears, it needs to be quickly identified. This is a challenge for underwater optical target recognition systems. In general, the complexity of a model is directly proportional to its accuracy: the larger and more complex the model, the higher its accuracy and the slower the corresponding detection speed. It is very important for underwater optical target recognition systems that the size of the model is compressed and its computational complexity is reduced whilst maintaining its detection accuracy.

With the rapid development of deep learning, underwater target recognition technology has made significant progress, leading to an increased algorithm accuracy, an enhanced robustness, an improved generalization ability, an optimized computational efficiency, the ability to adapt to changeable environments, and automatic processing in underwater target recognition tasks. To improve the underwater target recognition accuracy, many methods have been proposed, such as taking advantage of new models and technologies [3,4,5], data augmentation [6], feature augmentation, model parameter fine-tuning, multi-scale feature fusion, loss function improvement [7], and training strategy optimization. The introduction of new models often brings about a significant leap in performance compared to other methods. Recognition models can generally be categorized into traditional methods, two-stage approaches, and single-stage approaches. Traditional image processing techniques involve complex computations during the feature extraction phase and tend to have lower accuracy. Two-stage recognition methods offer higher precision but come with higher computational complexity, which, in underwater environments, leads to substantial consumption of computational resources. The inference speed of complex models is slow, further constrained by the limitations of underwater hardware resources. Existing recognition methods are susceptible to variations in underwater lighting conditions and contrast, interference from complex backgrounds, and the multi-scale nature of underwater targets, which can all lead to a decrease in recognition accuracy. These limitations make them less suitable for underwater target recognition systems. YOLO (You Only Look Once) is a popular single-stage deep-learning object-detection algorithm known for its real-time performance, accuracy, and ease of use. Since it was first proposed in 2015, YOLO has gone through multiple iterations to continuously improve its performance and applicability. It has been widely studied and applied in computer vision tasks; the latest proposed versions are YOLOv9 [4] and YOLOv10 [5]. As a large model, YOLOv9 requires more computing resources and is not compatible with devices with limited resources. Compared with YOLOv8, YOLOv10 has been significantly improved and optimized in the field of object detection; the removal of non-maximal suppression (NMS) has resulted in an increase in speed, and it continues to play an important role in eliminating overlapping detection frames and improving the accuracy of detection results. However, its detection capability may be slightly inferior to that of YOLOv8, especially for very small or heavily occluded targets. YOLOv8 achieves a favorable balance between model complexity and inference speed. It is designed to be more lightweight, making it suitable for operation in resource-constrained underwater environments. YOLOv8 boasts enhanced compatibility, allowing for easier deployment and execution across various hardware platforms. The YOLOv8 series is well established and stable, and its accuracy has been significantly improved on the foundation of YOLOv5, being widely adopted in the detection of underwater targets [8]. This includes the lightweight YOLOv8 model, an enhanced YOLOv8 model specifically optimized for underwater scenarios and the detection of dark underwater targets, as well as image blur and small target detection.

Aiming to solve the problems of a high computational complexity, slow detection speed and low accuracy present in underwater target detection models, the underwater target detection method proposed in this study (SPSM-YOLOv8) is a lighter optimization of YOLOv8n, making it more suitable for the real-time detection of underwater targets. Firstly, to enhance computational efficiency, we replace the 2D convolutions in the original YOLOv8n backbone network with lightweight SPDConv modules and to enhance the feature representation, we focus on the interaction between pixels. We have also integrated the PSA in YOLOv10 into YOLOv8n. Secondly, to avoid sacrificing the accuracy of the model and effectively reduce the computational cost and the number of parameters, we use the SCDown method. Finally, the MpdIoU is used to replace the CIoU loss function to accelerate the convergence speed of the bounding box and improve the accuracy of the bounding box regression.

The remainder of this manuscript is organized as follows: Section 2 describes the work, Section 3 describes the structure of the network, the results of the experiment are given in Section 4, and the conclusions are given in Section 5.

## 2. Model-Related Work

### 2.1. YOLOv8 Introduction

**Figure 1:**
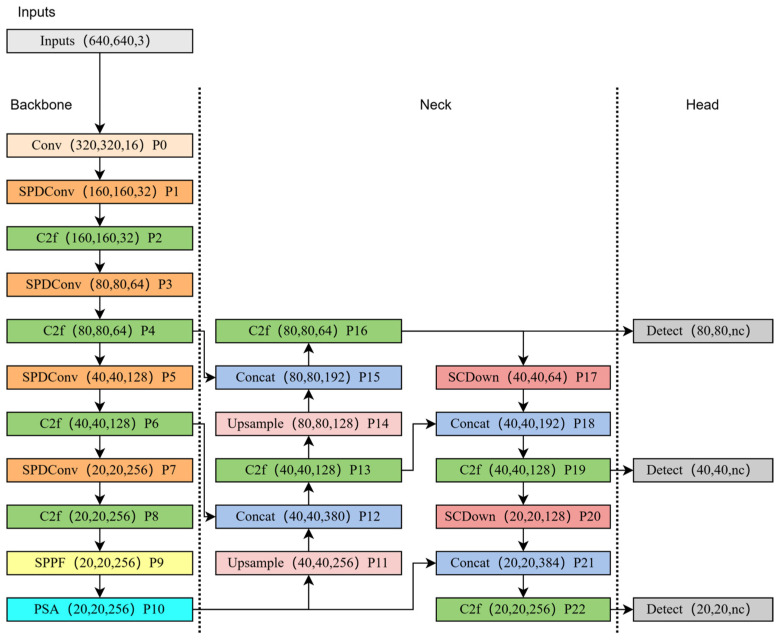
Structure of YOLOv8n. SPDConv, PSA, and SCDown network structures are added.

Launched by Ultralytics, YOLOv8 is one of the most advanced YOLO series models available. YOLOv8 inherits and optimizes the previous features of the YOLO series and introduces several innovative technologies and improvements to further improve performance and applicability: YOLOv8 uses a new C2f module, replacing the C3 module in YOLOv5. This module not only reduces the amount of computation but also improves the speed and convergence effect. The structure of YOLOv8 was transformed from an anchor-based to an anchor-free detection head structure, which allows for direct prediction of the center of the object, simplifies the number of box predictions, and accelerates the non-maximal suppression (NMS) process. YOLOv8 adopts the Task-Aligned Assigner strategy, which abandons the previous IoU allocation or unilateral proportional allocation method and further improves the accuracy of label allocation. YOLOv8 has an optimized PAN structure in the neck part; the 1 × 1 downsampling layer is removed, and the number of parameters and the overall size of tensors are reduced. In addition, YOLOv8 not only supports object detection tasks but also extends to instance segmentation and image classification. The series comprises pre-trained models at different scales, including N, S, M, L, and X, which are suitable for different application scenarios and deployment platforms. In this paper, YOLOv8n is used as the baseline network to achieve a lightweight and high-accuracy underwater target detection model. Four major modifications have been made to YOLOv8n, and the overall architecture of the modified SPSM-YOLOv8 network is shown in Figure 1.

### 2.2. Underwater Object Detection

The proposed underwater target detection method is divided into one- and two-stage methods. Two-stage methods, for example, RCNN and Faster-RCNN [9], use the regional suggestion module to generate candidate target suggestions and then classify and regress the candidate target suggestions. On this basis, many scholars have identified underwater targets and improved the network performance. Song et al. [10] proposed a two-stage underwater detector with enhanced R-CNN. The introduction of a new regional proposal network, a probabilistic inference pipeline, and a hard example mining method effectively improves the accuracy and reliability of object detection in complex underwater environments. Yi et al. [11] proposed a coordinate-aware Mask R-CNN, which combines object coordinate information and image enhancement technology to improve the accuracy and selectivity of detection and thus reduce the damage to marine ecosystems caused by unsustainable fishing activities. Two-stage algorithms for underwater detection offer high precision but require more computational resources, resulting in slower detection speeds and larger model sizes.

Single-stage methods can be further divided into Transformer-based methods and CNN-based methods. With its applications growing, Transformer has also made significant progress in the field of computer vision, providing new solutions, such as RE-DETR (Real-Time DEtection TRansformer), to solve complex vision problems through its unique attention mechanism. Wang et al. [12] proposed DyFishNet to extract fish texture features and fuse fish body feature information. The mAP in the underwater dataset reached 99%. CNN-based detection methods include SSD (Single-Shot MultiBox Detector) [13], YOLO, CenterNet [14], and RetinaNet. CNNs’ powerful feature extraction capabilities have achieved remarkable results regarding improving the detection accuracy and efficiency and have been utilized in many studies on underwater target recognition. Wang et al. [15] used depth-separable convolution to construct a lightweight SSD detection model to improve the efficiency and accuracy of lobster farming and reduce operating costs. Yuan et al. [16] comprehensively evaluated the performance of YOLOv5 and DETR for sea cucumber detection in a turbid underwater environment in terms of accuracy, real-time performance, and weight. Zhu et al. [17] integrated the EMA attention mechanism into YOLOv8 to simplify the calculation and improve the accuracy of underwater garbage detection. In summary, single-stage algorithms require less computational power and offer faster inference times. They have fewer parameters and a smaller model size, which translates to better real-time performance in underwater applications. These methods can balance precision and speed in complex underwater environments, a feat that two-stage algorithms often struggle to achieve. However, the aforementioned approaches typically focus on only one aspect of underwater detection models. Our method, based on YOLOv8n, maintains high precision while considering the speed of the detection model and reducing computational complexity. It meets the requirements of accuracy, speed, and lightweight design for embedded underwater target recognition systems.

## 3. Network Architecture

This section describes the principles and functions of each of the four improved modules. Firstly, the convolution of spatial depth transformation is introduced, and then the principles, advantages, and disadvantages of partial self-attention are discussed. The function of double downsampling of spatial channels is then introduced. Finally, the MPDIoU loss function is discussed.

### 3.1. Space-to-Depth Convolutions (SPDConv)

**Figure 2:**
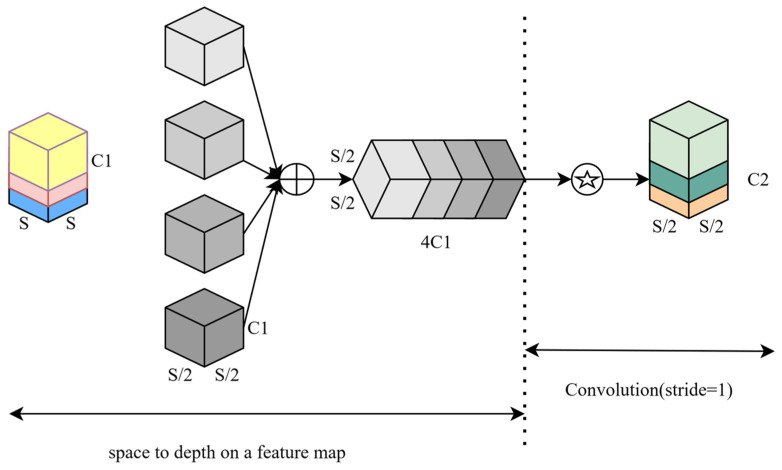
SPDConv network structure. In Figure 2, the “⊕” operation signifies the concatenation of different feature groups along the channel dimension, while the operation denoted by the circled asterisk applies a convolution with a stride of 1 to the feature map.

In recent years, with the continuous advancement of deep learning, object detection models have achieved remarkable results and are widely applied in various fields. However, large models are not suitable for real-time visual tasks with limited computational resources, such as underwater target recognition. Therefore, lightweight network solutions are needed. Mainstream lightweight network models include MobileNetv3 [18], ShuffleNetv2 [19], GhostNet, EfficientNetLite, etc. They construct different lightweight structures through depthwise separable convolution, significantly reducing computation and model parameters, thus improving efficiency, as seen in the literature [20,21,22]. Traditional convolutional layers, comprising stride convolutions and pooling layers, are designed to reduce the spatial dimensions of feature maps and expand the receptive field. However, in deep networks, each convolutional layer performs a substantial number of convolution operations on the input, leading to an increase in computational complexity. To address these limitations, the Spatial Depth-Wise Transform Convolution has been introduced [23]. SPDConv represents an innovative spatial encoding technique that replaces stride convolutions and pooling layers in CNNs. It employs Spatial-to-Depth (SPD) layers to transform the spatial dimensions of input feature maps into depth dimensions, thereby increasing the number of channels to preserve more information. Thanks to its unique feature extraction approach, SPDConv enhances computational efficiency under limited computational resources. Specifically, following the SPD layer, a convolutional layer with a stride of 1 is used to reduce the number of channels while processing features with learnable parameters. By eliminating stride convolutions and pooling layers, SPDConv reduces information loss. In resource-constrained edge computing systems underwater, SPDConv improves the efficiency of feature extraction, reduces computational complexity, and accelerates model operation. SPDConv is capable of converting spatial features into depth features, capturing higher-level semantic information, which aids in enhancing the model’s ability to recognize underwater targets. This achieves greater perceptual power and improves the model’s recognition accuracy. The network structure of SPDConv is illustrated in Figure 2.

### 3.2. Partial Self-Attention (PSA)

Underwater backgrounds are complex and varied, with target sizes ranging widely. Most underwater targets fall within the 128 × 128 pixel range, with the highest concentration of targets being within the 64 × 64 pixel range; object detection algorithms often use attention mechanisms to selectively focus on key parts so that the model can better understand and process complex sequence data. However, the attention mechanism is very computationally complex and occupies a large amount of memory when processing high-resolution images. Choosing an attention mechanism that can enhance the representation of model features and that has a high computational efficiency is crucial for the accuracy and real-time performance of the end-to-end detection model. The partial self-attention (PSA) [5] attention mechanism has been introduced into the YOLOv8 backbone network, allowing the model to focus on the key feature information of the underwater target when processing a large amount of information and to improve the processing efficiency and accuracy. In previous research, attention mechanisms were mainly categorized as spatial attention, channel attention, and self-attention mechanisms, all of which have some shortcomings. Spatial attention can improve local information perception and adjust the focus to adapt to changes in different scales and resolutions. However, the computational resource consumption is high, and the generalization ability is limited, which is evident in deformable convolution DCNv4 [24]. By redistributing the weights of different channels, channel attention enhances the feature representation and accuracy of the model, and its design is relatively simple. However, an over-reliance on precise channel weights leads to the loss of spatial information, for example, in SENet [25] and CBAM. Spatial-and-channel attention considers both spatial and channel characteristics, but there are limitations with regard to capturing long-distance dependencies, and the computational complexity is high, thus affecting the model speed. The self-attention mechanism considers global contextual information and has strong long-distance dependence and adaptability. However, computing resources are expensive and require a large number of raw data, for example, in self-attention [26]. The PSA mechanism introduced in this study integrates global and local information and solves the problem of large computational costs and long-distance dependence on the self-attention mechanism, thus improving network performance.

**Figure 3:**
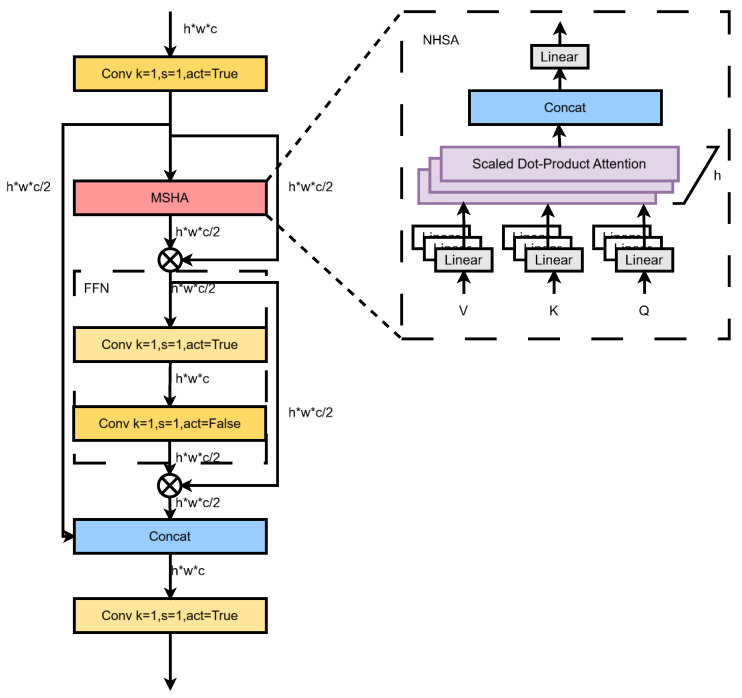
PSA network structure. In Figure 3, the “⊗” symbol denotes the operation of tensor multiplication.

The partial self-attention mechanism divides the feature map into different scales, performs self-attention calculations on these different scales, and then synthesizes the calculation results of these parts to form the final output. This method can enhance the model’s perception of local and global features at a low computational cost, and, at the same time, it has the advantage of long-distance dependence on self-attention. The process structure is shown in Figure 3. First, the features are evenly divided into two branches via 1 × 1 convolution. Only a portion of them is then fed into an NPSA block consisting of a multi-head self-attention module (MHSA) and a feedforward network (FFN). Finally, the two parts of the features are spliced together, and the number of channels is fused via 1 × 1 convolution. The multi-head self-attention module and the feedforward network are derived from the Transformer [27]. Dividing the model into multiple headers to form multiple subspaces allows the model to focus on different aspects of information. The MHSA projects the input to h lower dimensions, calculates h attention, concatenates the output of h attention in the feature dimension, and then finally uses MLP to perform multi-head feature aggregation to obtain the output of MHSA. The FFN layer is actually a linear transformation layer that is used to complete the dimensional transformation of the input data to the output data. In this study, after the FFN layer, the number of channels changes from C/2 to C and then to C/2, ensuring that the input and output dimensions of the FFN are consistent, enhancing the feature extraction ability and computational efficiency of the model.

### 3.3. Spatial–Channel Decoupled Downsampling (SCDown)

**Figure 4:**
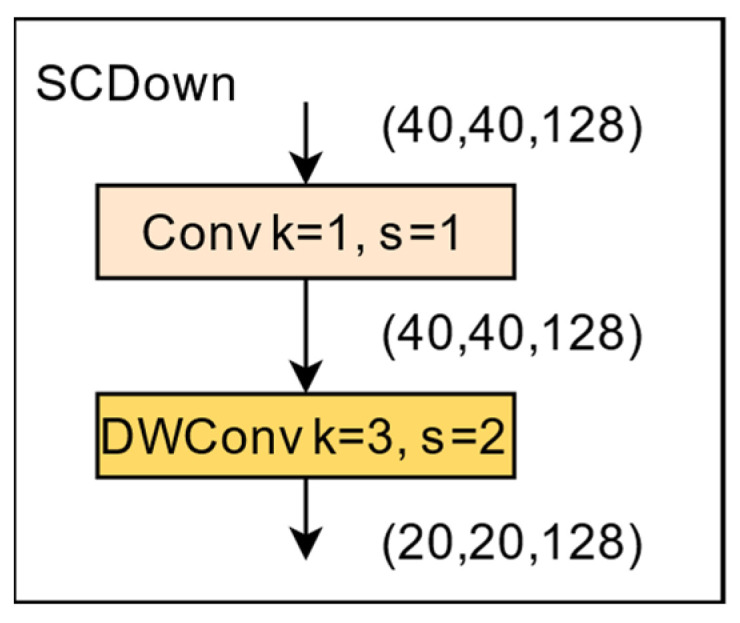
SCDown network structure.

In image processing, downsampling is an effective means to reduce the image size and computational cost while retaining key information. Traditional downsampling methods usually use larger convolutional kernels directly but increase the amount of computation and the number of parameters. Proposed in YOLOv10 [5], SCDown significantly reduces the treatment of both by separating space and channels. By using point convolution, the channel dimensions of the input feature map are adjusted, effectively retaining important feature information and reducing the amount of computation. Deep convolution is then used for spatial downsampling. Deep convolution effectively reduces the spatial dimension of the feature map, retains the downsampling information to the greatest extent, and maintains the detection accuracy of the model, but it does not increase the additional computational cost. Specifically, SCDown sampling utilizes 1 × 1 convolution to adjust the channel dimension and then uses deep convolution (3 × 3 convolution) for spatial downsampling. This process effectively reduces the cost of calculations while preserving more information, which helps to improve the accuracy and speed of object detection. Figure 4 shows the structure of the SCDown network.

### 3.4. MPDIoU Loss Function

**Figure 5:**
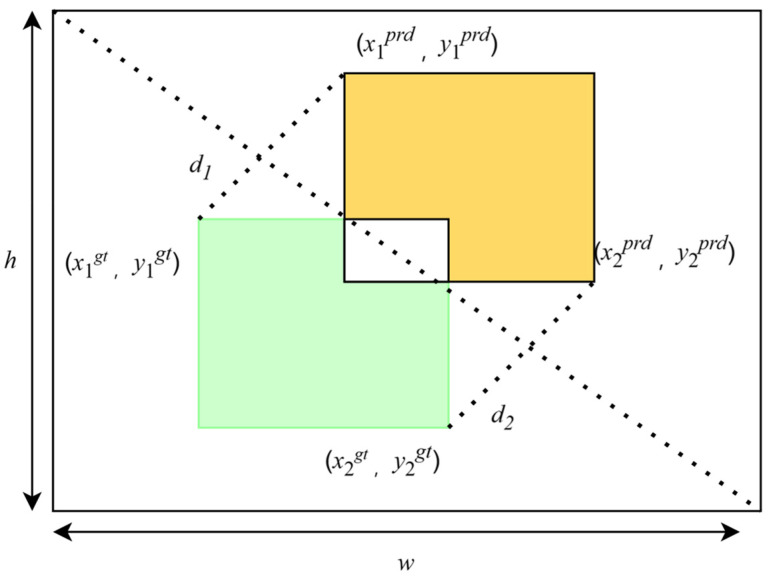
Illustration of MPDIoU calculation.

The loss function is a metric used to quantify the discrepancy between a model’s predicted values and the actual observed values. The goal is to minimize this loss, ensuring that the model’s predictions closely align with the actual outcomes. The original loss function of Yolov8, CIoU, takes into account the overlapping area between bounding boxes, as well as the distance between center points and the aspect ratio, making it more effective in dealing with objects of varying shapes and sizes. However, the computation of the CIoU loss function is relatively complex due to the need to consider multiple factors, such as the overlapping area, center point distance, and aspect ratio simultaneously. This complexity leads to higher computational costs. CIoU is highly sensitive to differences in aspect ratios, causing the model to focus excessively on aspect ratio matching during training, while neglecting other important geometric factors. Its computational complexity and sensitivity to aspect ratios can, to some extent, hinder the model’s convergence speed. To address these issues, MpdIoU [7] was proposed. MpdIoU simplifies the calculation process by using the minimum point distance as a metric for bounding box similarity, thereby enhancing accuracy. By introducing point distance optimization, MPDIoU effectively resolves the optimization problem where predicted bounding boxes have the same aspect ratio as the true bounding boxes but differ significantly in width and height, thus improving the model’s performance in complex scenarios. Additionally, MPDIoU reduces the computational load, allowing for faster model training. MPDIoU builds upon CIoU with several improvements, enhancing the model’s accuracy and training speed in bounding box regression tasks. In underwater target recognition, MPDIoU more accurately reflects the differences between bounding boxes, aiding the model in pinpointing underwater targets with greater precision. MPDIoU better adapts to changes in the shape of underwater targets, improving the model’s robustness and accuracy. Compared to traditional IoU, MPDIoU reduces computational complexity. The calculation of MpdIoU is illustrated in Figure 5.

The calculation is as follows:

(1) $$IoU=\frac{A\cap B}{A\cup B},$$

(2) $$d_{1}^{2}={\left(x_{1}^{prd}-x_{1}^{gt}\right)}^{2}+{\left(y_{1}^{prd}-y_{1}^{gt}\right)}^{2},$$

(3) $$d_{2}^{2}={\left(x_{2}^{prd}-x_{2}^{gt}\right)}^{2}+{\left(y_{2}^{prd}-y_{2}^{gt}\right)}^{2},$$

(4) $$MPDIoU=IoU-\frac{d_{1}^{2}}{w^{2}+h^{2}}-\frac{d_{2}^{2}}{w^{2}+h^{2}},$$

where IoU is used to describe the degree of coincidence between two boxes. The numerator is the intersection of the two boxes, and the denominator is the union of the two boxes, so their ratio represents the intersection and union ratio; $x^{prd}$ and $y^{prd}$ represent the upper-left and lower-right point coordinates of the prediction box, respectively; $x^{gt}$ and $y^{gt}$ represent the upper-left and lower-right point coordinates of the true box, respectively; and d represents the distance to the coordinate point.

## 4. Experiments

### 4.1. Datasets and Estimated Metrics

In this study, two publicly available underwater datasets were used to validate the proposed SPSM-YOLOv8 detection method. The details of the datasets are as follows:

RUOD [28]: RUOD is an underwater dataset for unmanned robot operation, providing rich image data and annotation information. Three environmental challenge test sets were also designed, namely the fog effect, color shift, and light interference. The addition of these special scenarios makes the dataset more comprehensive and can effectively allow us to evaluate the robustness and accuracy of the detection algorithm in complex environments. The dataset contains a total of 13,950 images, and the resolution of the images is 1920 × 1080 pixels. There are 10 types of underwater targets: holothurian, echinus, scallops, starfish, fish, corals, divers, cuttlefish, turtles, and jellyfish. Two non-intersecting subsets of the dataset were used as the training and test sets, containing 9800 and 4150 images, respectively. A total of 1900 images were randomly selected from the training set as the validation set.

URPC2020 [29]: The dataset from the underwater optics group of the Dalian Underwater Target Detection Algorithm contains 5543 underwater optical images, which are divided into a training set, a verification set, and a test set according to a 6:2:2 ratio. The image resolution is 1920 × 1080 pixels. There are four types of detection targets: sea cucumbers, sea urchins, starfish, and scallops.

The detection accuracy is measured using precision, recall, and average precision (mAP). Precision represents the proportion of samples that are predicted to be positive that are actually positive. Recall indicates that the prediction result is the proportion of the actual number of positive samples in the positive sample to the positive sample in the whole sample. mAP represents the mean of the average precision; the accuracy of each category is predicted, and then the sum is divided by the total number of categories.

The calculation is as follows:

(5) $$Precision=\frac{TP}{TP+FP},$$

(6) $$Recall=\frac{TP}{TP+FN},$$

(7) $$AP=\int_{0}^{1}precision(t)dt,$$

(8) $$mAP=\frac{\sum_{n=1}^{N}AP_{n}}{N},$$

where TP is a correct target sample for detection, FP is a sample that is falsely identified as the target, and FN is a correct target sample that is not identified as the target.

Frame rate (FPS) is a measure of the speed of the model, which is the reciprocal of the time it takes to process the image, with a higher FPS indicating faster processing. The model parameters are the sums of all trainable weights and biases in the model, and they are constantly adjusted during model training to minimize the loss function. Floating-point operations (GFLOPs) represent the number of floating-point operations required in the execution of an algorithm or model and are important indicators to measure the complexity of the algorithm and hardware requirements, which are directly related to computing speed and resource consumption. The model size usually reflects the number of model parameters, including weights and biases, which directly affect the storage requirements and computational complexity of the model.

### 4.2. Experimental Equipment and Parameter Configuration

All experiments in this study were conducted on Windows 11, using the PyTorch 2.0.1 framework, it is equipped with an RTX 3070TI 8G memory from NVIDIA Corporation in the Santa Clara, CA, USA and a 12th Gen Intel(R) Core(TM) i5-12490F 3.00 GHz CPU from Intel Corporation in the Santa Clara, CA, USA. YOLOv8n was used as the baseline, and the pre-trained model was not used. The stochastic gradient descent optimizer was used for training on 150 epochs. The initial learning rate was 0.01, the initial pulse was 0.937, the weight decay was 0.0005, and the input image size was 640 × 640. The training batch size was 4. When calculating the FPS, the GPU was first warmed up, and a total of 10 tests were performed to remove the longest and shortest inference times, which were used to remove the influence of the hardware and derive the FPS of the model. The IoU threshold for non-maximum suppression was set to 0.30, and the confidence level was set to 0.40.

### 4.3. Comparative Experiments on the RUOD Dataset

**Figure 6:**
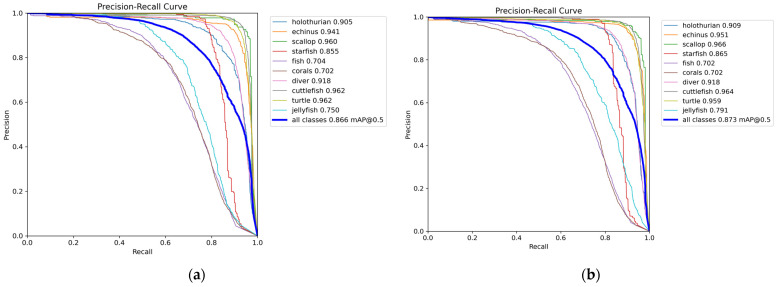
Precision–recall curves for the models: (**a**) YOLOv8n and (**b**) SPSM-YOLOv8.

To verify the effectiveness of the SPSM-YOLOv8 algorithm in this study, we chose to compare its performance on the RUOD dataset with that of other State-of-the-Art models. The models’ results (in bold) were derived from the experiments in this study. The improved algorithm was evaluated by model size, floating-point operations, number of parameters, average accuracy of the algorithm, and FPS, and a comparison is shown in Table 1. The highest average accuracy is for YOLOv9c, reaching 90.3%, but the number of parameters and model size are too large, which is not suitable for underwater embedded edge computing systems that are sensitive to computing resources. The lowest number of parameters is for YOLOv5n, but its average accuracy is only 86.4%. The SPSM-YOLOv8 model proposed in this paper has an average accuracy of 87.3%, a parameter quantity of 2.87 M, a model size of 6.0 M, 7.7 G floating-point operations, and an FPS of 189 f/s. Figure 6 shows the P-R curves for the SPSM-YOLOv8 model. Compared with the baseline model (YOLOv8n), the mAP is increased by 0.7%, the number of parameters is reduced by 4.3%, and the floating-point operations are reduced by 4.9%. The SPSM-YOLOv8 model is 3.8%, 0.9%, 8.6%, 1.1%, and 0.9% more accurate than the classic YOLOv3-tiny, YOLOv5n, YOLOv6, YOLOv7-tiny, and YOLOv10 models, respectively. Compared with YOLOv3, YOLOv5m, YOLOv7, YOLOv9c, and RT-DETR, the floating-point operations are reduced by 147 G, 40.3 G, 97.6 G, 94.7 G, and 29.9 G, respectively. The model size was reduced by 117.6 M, 36.2 M, 68.9 M, 45.6 M, and 25.5 M, respectively. A comprehensive analysis proves that the improved algorithm for underwater garbage recognition is better than commonly used object detection algorithms; it achieves the highest average accuracy among all algorithms, and it has the lowest floating-point operations and smallest parameter model. It can thus be deployed on mobile or embedded devices and can effectively and accurately realize underwater target detection.

### 4.4. Convergence Analysis

**Figure 7:**
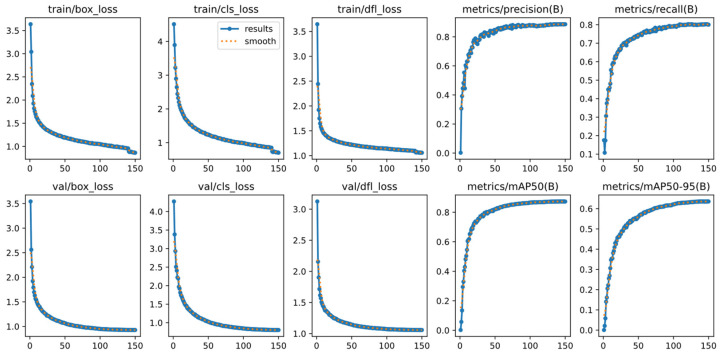
Training-process curves.

Comparing the training process curves in Figure 7, it is clear that the bounding box loss, classification loss, and distribution focus loss convergence of the training process and the verification process have improved. The model improved rapidly in terms of accuracy, recall, and average accuracy, leveling off after about 100 epochs, and training was stopped at 150 epochs.

### 4.5. Improving the Performance of the Backbone Network

Table 2 shows the detection effect of the improved backbone network on the RUOD dataset compared with that of the commonly used backbone methods. The performance results for the models (in bold) are derived from the experiments in this study. The model performance analysis (mAP_50_%) shows great variety across the different models, ranging from the lowest (83%) to the highest (88.9%). EfficientNetV2, FasterNeT, RepViT [31], and MobileNetV3 achieved a high mAP_50_% value. Compared with the improved CSP (SPDConv) networks in this study, the number of parameters and the computational complexity are significantly increased, and none of the FPSs exceeded 200. In terms of model complexity (parameters and model size), lightweight networks such as MobileViTv2, ShuffleNetV2, and GhostNet and high-efficiency networks such as ResNet series and PP-HGNetV2 [3] are relatively non-complex, making them suitable for deployment on resource-constrained devices. Models such as LSKNet, SwinTransformer, and VanillaNet [32] have more parameters and are larger; thus, they may require more powerful computing resources. In addition, their accuracy is inferior to that of the improved network proposed in this study. From the computational efficiency analysis (GFLOPs and FPS), high-efficiency models such as ResNet series, MobileNetV4 [33], SCINet [34], SENetV2, and CSP have lower GFLOPs and a higher FPS, indicating that they are more computationally efficient. The improved CSP (SPDConv) in this paper has a higher mAP_50_%. In general, the improved CSP (SPDConv) backbone network presented in this paper achieved good results regarding three aspects: model performance, model complexity, and computational efficiency. In particular, in terms of model complexity, the number of model parameters is reduced by 10%, and the number of floating-point operations is reduced by 7%. At the same time, the FPS remains high. Thus, the model is suitable for real-time detection in underwater environments. However, compared to YOLOv8n, the detection accuracy decreased slightly; because SPDConv is mainly used for local feature extraction, it lacks spatial downsampling capability. When dealing with tasks on different scales, additional downsampling modules are thus required to implement multi-scale representations.

### 4.6. Impact of Different Attention Mechanisms

In this study, we added common attention mechanisms such as ECA, CA, GAM, and CBAM to the backbone network; other attention mechanisms have been studied in the literature [49,50,51,52,53,54,55,56,57,58,59,60,61,62,63]. Eighteen sets of comparative experiments were designed to compare the performance and accuracy of the model after adding each attention mechanism to the baseline model (the results are shown in Table 3). Comparing the 18 attention mechanisms added, we see that the PSA mechanism has the highest accuracy, delivering an improvement of about 0.9%. The model complexity remains largely the same, with an FPS of 226 frames per second. MLCA [59] has the lowest recognition accuracy, and CGA [43] has the lowest detection accuracy, which is lower than that of the baseline model. In terms of computational complexity, there is a non-significant increase in model complexity for several other attention mechanisms. HAttention [64] has the largest number of parameters and is not suitable for systems with limited computing resources. In terms of detection speed, CBAM, LSKA, and GAM achieved more than 250 frames per second. However, their accuracy is not as high as that of PSA.

**Figure 8:**
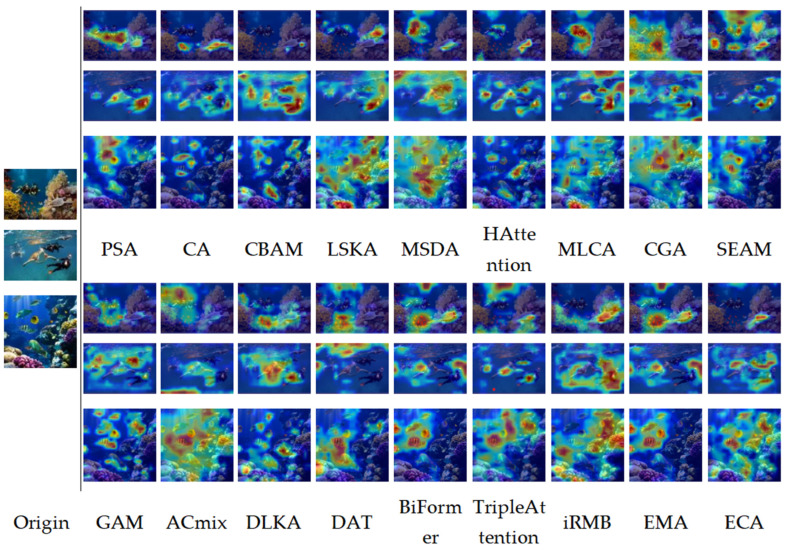
Comparison of heatmap effects of each attention mechanism.

In addition, we used Grad-CAM to create a heat map of the original model and the model after adding the attention module to visualize the key areas of network interest in underwater target recognition (the results are shown in Figure 8). The visualized results in the figure are all P9 layers of the detection model. A qualitative analysis of the heatmap images shows that PSA can accurately focus on target features of different sizes in the background, which is significantly superior to other attention mechanisms. It is noteworthy that PSA has enhanced its global information learning capabilities, resulting in an overall performance improvement at multiple scales. Overall, adding an attention mechanism leads to a certain improvement in accuracy. In the detection of large targets, PSA, LSKA, MSDA, and HAttens exhibit a better performance, while MLCA and CGA failed in the detection process. When detecting multiple targets, PSA, HAttention, and SEAM yield a better performance, while LSKA, MSDA, MLCA, and CGA focus on the communication area between targets. When detecting complex background targets, PSA and HAttention have good performances, while SEAM is affected by the complexity of the background and focuses on many irrelevant background regions. According to the quantitative analysis of the model evaluation results in Table 3 and Figure 8, PSA has the highest accuracy, CGA has the worst performance, and HAttention has the highest computational complexity. Based on the results of the quantitative and qualitative analysis, we added the PSA module after the SPPF module. In later experiments, it was also proven that the addition of the PSA module had a greater improvement effect on the model.

### 4.7. Comparison of Loss Functions

To verify the influence of the added MPDIoU loss function on the accuracy of the model, different bounding box loss functions were added to the original model and compared, and the experimental results are shown in Table 4. CIoU (Complete-IoU) is a loss function used to measure the similarity between a predicted and real box in the field of object detection. CIoU not only considers the overlapping areas of the two bounding boxes, but it also introduces a correction factor to more accurately measure the similarity between the target boxes. DIoU (Distance-IoU) improves the target box regression stability by considering the distance, overlap rate, and scale between two bounding boxes. EIoU (Efficient-IoU) additionally considers the distance between the center point, the difference in scale, and the minimum size of the closed box based on the traditional IoU so that the loss function can more sensitively reflect the target’s position and shape changes. SIoU (Soft-IoU) is a soft-threshold-based IOU method which introduces a soft threshold function to force the IOU calculation result to consider the fuzzy boundary between the target boxes. Weighted Intersection over Union (WIoU) effectively improves the performance of the object detection model by introducing a weight factor and a dynamic non-monotonic focusing mechanism. Shape-IoU is a loss function used to measure the similarity of shapes between a predicted box and a real box. Shape-IoU provides a more accurate and robust way to do this by considering factors such as the overlapping area of the bounding box, the distance between the center point, and the aspect ratio. MPDIoU considers the scale difference between the bounding boxes, which can more accurately reflect the matching degree between the prediction box and the real box and improve the positioning performance of the model. Compared to other loss functions, the average accuracy is the highest.

### 4.8. Ablation Experiments

**Figure 9:**
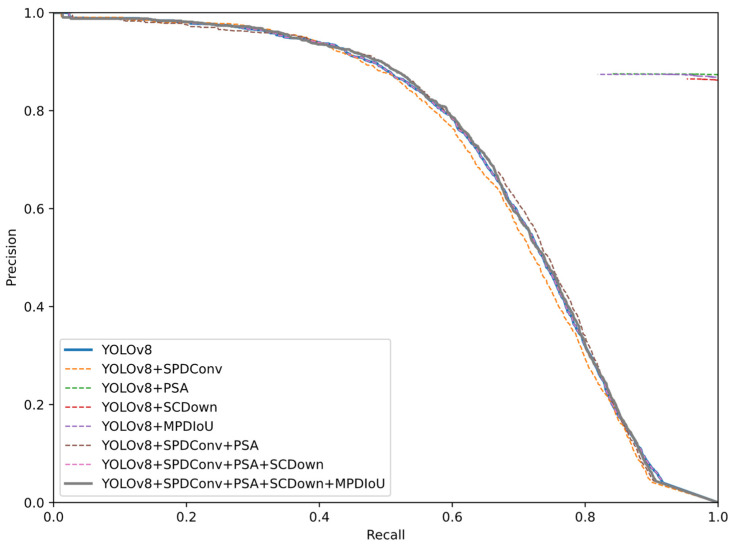
P-R curves.

To verify the impact of the added improved modules on the overall model accuracy and the impact of the coupling between modules on the performance, using YOLOv8n as the baseline model, ablation experiments with four improved modules were carried out on the RUOD dataset. The results of the ablation experiment are shown in Table 5. After adding SPDConv, PSA, SCDown, and MPDIoU to the baseline model, the model achieves the best accuracy of 87.3% (mAP_50_) for fish recognition in an underwater fuzzy scene, which is 0.7% higher than the original detection model. The number of parameters is reduced by 0.13 M, the model size is reduced by 0.2 MB, and the floating-point arithmetic is reduced by 0.4 G. After adding the SPDConv module, the number of parameters is reduced by 10.6% and the number of floating-point operations is reduced by 0.6 G. This indicates that SPDConv rearranges the spatial information of the feature map to the depth/channel dimension, reducing the spatial size of the feature map. This reduces the amount of computation required in the future. A stepless convolution operation is added after each SPD layer to reduce the number of channels. With the addition of the PSA mechanism, the average accuracy of mAP_50_ and mAP_50-90_ increased by 0.9% and 0.8%, respectively. The number of parameters increased by 0.12 M, and the fps decreased by 34. This shows that PSA can improve the accuracy of object detection by introducing the self-attention mechanism to enable the model to capture the global information in the image more effectively. However, it is also relatively computationally expensive. After adding the SCDown attention mechanism, the number of parameters decreased by 0.16 M, and the model size decreased by 0.3 MB. This shows that SCDown adjusts the number of channels through (1 × 1) point convolution and then uses deep convolution to reduce the spatial dimension for downsampling. This approach reduces the consumption of computational resources while maintaining model performance. After adding the MPDIoU loss function, the computational complexity of the model does not change, but the mAP_50_ is increased by 0.8%. The results show that MPDIoU considers the similarity between the prediction frame and the real frame and significantly improves the detection accuracy. The improved SPSM-YOLOv8 model has the best overall performance and the lowest complexity on the RUOD dataset, which is conducive to the application of underwater edge computing devices. The PR curves of the model following the ablation experiment are shown in Figure 9. The ablation experiments on the URPC2020 dataset also verify the superiority of the SPSM-YOLOv8 algorithm.

### 4.9. Experiment Validation

**Figure 10:**
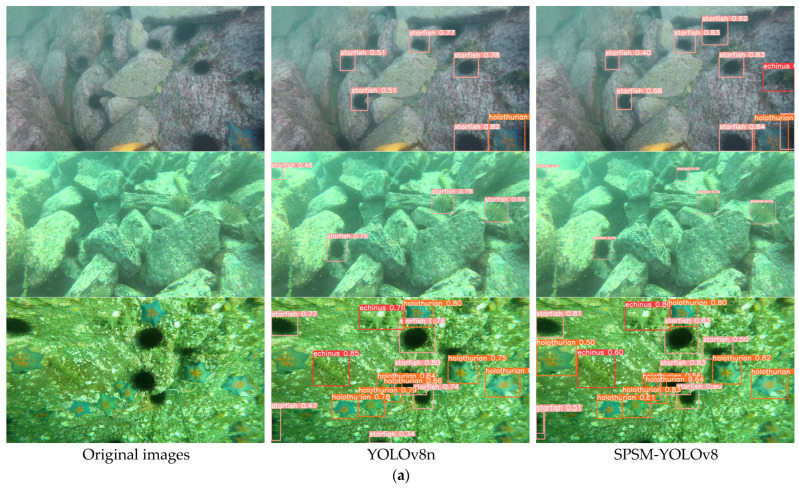

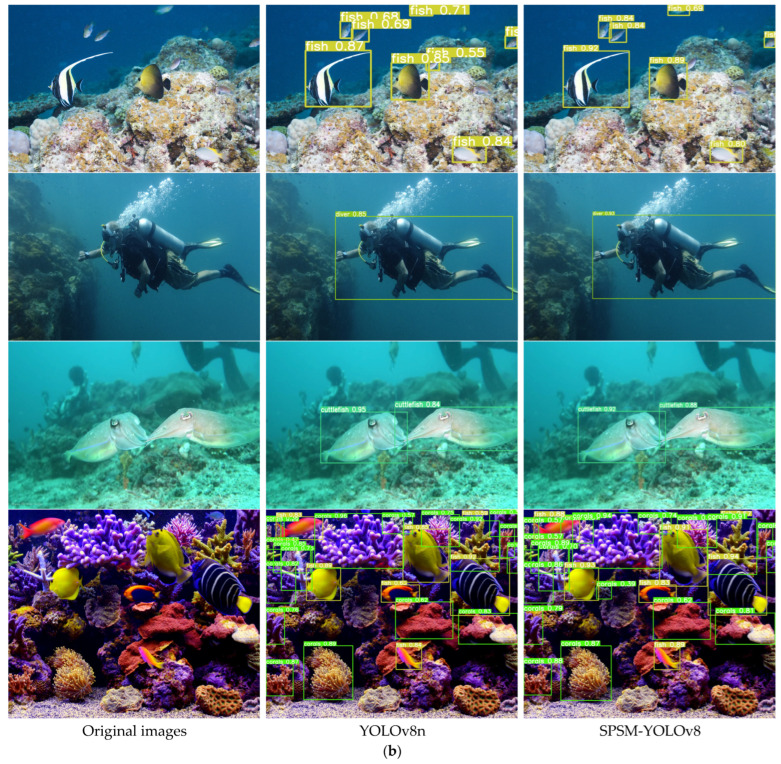
Comparison of the detection performance of SPSM-YOLOv8 and YOLOv8n. (**a**) Comparison of detection performance on URPC2020 datasets. (**b**) Comparison of detection performance on the RUOD dataset.

To further verify the effectiveness of the proposed algorithm, we deployed it on both a PC and Jetson Xavier Nx, and targets from the URPC2020 and RUOD datasets were predicted. The control random seed and model fine-tuning parameters were consistent, and the detection results were consistent between PC and Jetson Xavier Nx (the results are shown in Figure 10). When compared on the URPC2020 dataset, YOLOv8n tends to miss detections when the color of the starfish closely resembles the background. The SPSM-YOLOv8 model detects more target boxes and exhibits higher regression accuracy within those boxes compared to YOLOv8n. This indicates that SPSM-YOLOv8 outperforms YOLOv8n in detecting underwater targets with similar colors to the background, demonstrating greater adaptability to the background and, consequently, higher precision. On the RUOD dataset, it is evident that SPSM-YOLOv8 achieves superior detection results compared to the baseline YOLOv8n. Across both datasets, the experiments suggest that SPSM-YOLOv8 possesses robust generalization capabilities. With higher recognition accuracy than YOLOv8n, SPSM-YOLOv8 is more suitable for underwater detection tasks.

## 5. Conclusions

This study proposes a lightweight real-time detection network model that considers the complexity of underwater environments. Taking YOLOv8n as the baseline model, we first replace the standard convolutional modules in the backbone network with SPDConv modules for feature extraction. This enhances computational efficiency and reduces redundant computations. Secondly, after the SPPF module, the PSA mechanism is added. Polarization filtering and enhancement of features in the channel and spatial dimensions are carried out to improve the pixel-level prediction accuracy. The SCDown downsampling module is introduced to decouple the space and channel operations and reduce the computing cost. Finally, MPDIoU replaces the original loss function to accelerate the convergence speed of the bounding box and improve the regression accuracy. The experimental results show that the proposed SPSM-YOLOv8 model has a high detection accuracy, a reduced size, a reduced number of computations, and a high FPS. The detection accuracy is maintained, while the size of the model is compressed and the computational complexity of the model is reduced, thus meeting the requirements of an underwater target detection algorithm that has high accuracy, is lightweight, and has fast edge deployment. However, this method still has some shortcomings and limitations. The proposed method requires adaptive adjustments for different visual tasks and cannot achieve cross-domain object detection from underwater imaging to natural imaging. Future work will continue to explore the potential of YOLO, addressing the scalability of underwater recognition models and their generalizability across various environments.

## Figures and Tables

**Table 1:** The performance of each detection model on the ROUD dataset.

Model	Precision	Recall	mAP_50_ (%)	Parameters (M)	Model Size (MB)	GFLOPs	FPS
Faster-RCNN	81.4	82.0	81.7	13.72	27.8	18.12	75.6
CenterNet	96.2	52.6	74.4	31.6	32.7	14.6	38.2
EfficientDet	84.2	53.4	63.7	5.9	6.6	6.1	183
YOLOv3-tiny	87.6	76.8	83.5	8.6	17.5	12.9	214
YOLOv3	90.4	84.1	88.6	61.5	123.6	154.7	54
YOLOv3	90.4	84.1	88.6	61.5	123.6	154.7	54
YOLOv5n	87.3	79.6	86.4	1.7	3.9	4.2	306
YOLOv5s	88.9	81.8	87.6	7.03	14.4	15.8	203
YOLOv5m	90.2	85.0	89.1	20.88	42.2	48.0	126
YOLOv5l	90.0	84.4	89.0	46.15	92.9	107.8	88
YOLOv6	82.9	71.0	78.7	4.23	8.7	11.8	276
YOLOv7-tiny	86.7	79.7	86.2	6.03	12.3	13.3	199
YOLOv7	88.4	85.3	89.6	37.24	74.9	105.3	77
YOLOv8n	88.0	79.1	86.6	3.00	6.2	8.1	260
YOLOv8s	88.7	83.7	89.0	11.12	22.5	28.5	208
YOLOv8m	89.4	84.9	89.6	25.84	52.0	78.7	109
YOLOv8l	89.8	85.1	89.4	43.61	87.7	164.9	53
YOLOv9c [4]	88.7	86.2	90.3	25.32	51.6	102.4	67
RT-DETR [3]	89.1	83.8	88.3	15.5	31.5	37.6	197
YOLOv10 [5]	86.2	80.2	86.4	2.69	5.8	8.2	246
PDSC-YOLOv8n [30]	85.5	81.2	86.1	5.62	11.4	8.96	297
**SPSM-YOLOv8 [ours]**	**88.6**	**80.1**	**87.3**	**2.87**	**6.0**	**7.7**	189

**Table 2:** Comparison of backbone networks.

Model	Backbone	mAP_50_ (%)	Parameters (M)	Model Size (MB)	GFLOPs	FPS
Baseline	CSP	86.6	3.00	6.2	8.1	260
	ResNet18 [35]	83.4	2.11	4.4	6.2	269
	ResNet34	83.9	2.18	4.6	6.4	258
	ResNet50	83.9	2.18	4.6	6.4	247
	ResNet101	84.4	2.63	5.5	7.9	193
	MobileViTv2 [36]	86.7	3.28	6.9	11.3	125
	MobileNetV3 [37]	87.2	5.65	11.7	9.4	158
	MobileNetV4 [33]	83.7	4.30	8.9	8.0	201
	ShuffleNetV2 [38]	85.6	2.79	5.9	7.4	153
	GhostNet [39]	84.9	5.35	11.2	8.2	104
	LSKNet [40]	83.5	12.31	24.9	36.6	87
	SCINet [34]	86.5	3.00	6.3	9.1	218
	SwinTransformer [41]	86.6	29.98	60.5	402.1	56
	SENetV2 [42]	86.7	3.09	6.4	8.2	232
	VanillaNet [32]	86.3	29.76	59.8	151.3	55
	RepViT [31]	87.2	4.12	8.6	11.7	96
	PP-HGNetV2 [3]	85.6	2.29	4.8	6.6	250
	EfficientViT [43]	86.1	4.01	8.7	9.4	55
	FasterNeT [44]	87.7	15.18	30.7	37.0	179
	EfficientNetV2 [45]	88.9	21.75	44.3	52.1	69
	RevColV1 [46]	83.0	32.09	64.6	79.3	51
	ConvNeXtV2 [47]	84.2	5.66	11.6	14.1	102
	UniRepLknet [48]	85.2	6.28	13.0	16.5	81
	EMO [49]	86.8	6.28	13.0	31.6	71
	**CSP (SPDConv)**	86.5	**2.68**	**5.6**	**7.5**	230

**Table 3:** The effect of adding different attention mechanisms.

Model	Method	Precision	Recall	mAP_50_ (%)	Parameters (M)	Model Size (MB)	GFLOPs	FPS
Baseline	Origin	88.0	79.1	86.6	3.00	6.2	8.1	260
	PSA [5]	88.6	81.4	87.5	3.12	6.5	8.1	226
	CA [53]	87.2	79.0	86.3	3.01	6.3	8.1	236
	CBAM [52]	87.5	79.7	86.1	3.07	6.4	8.1	268
	LSKA [58]	88.7	80.2	87.1	3.07	6.4	8.2	250
	MSDA [54]	88.7	79.7	87.0	3.27	6.8	8.3	193
	HAttention [64]	87.8	79.7	86.7	7.93	16.5	8.1	259
	MLCA [59]	87.9	80.3	87.3	3.00	6.3	8.2	203
	CGA [43]	86.6	78.8	86.1	3.05	6.7	8.3	74
	SEAM [63]	88.6	79.4	87.0	3.11	6.5	8.3	199
	GAM [57]	87.5	80.0	86.8	4.64	9.5	9.4	253
	ACmix [60]	88.9	79.8	87.1	3.30	6.9	8.7	117
	DLKA [50]	87.8	77.7	85.9	4.62	9.5	13.4	52
	DAT [62]	87.4	80.3	86.8	3.08	6.4	8.3	186
	BiFormer [51]	83.3	73.7	81.1	3.90	8.1	43.1	139
	TripletAttention [61]	88.2	78.6	86.5	3.00	8.1	6.3	169
	iRMB [49]	87.3	80.2	86.9	3.35	7.0	8.8	172
	EMA [56]	87.0	80.3	86.7	3.00	6.3	8.2	208
	ECA [55]	87.2	79.9	86.6	3.00	6.3	8.1	271

**Table 4:** Comparison of loss functions on URPC2020 dataset.

Model	Method	Precision	Recall	mAP_50_ (%)	mAP_50-90_ (%)	AP (%)			
						Echinus	Starfish	Holothurian	Scallop
Baseline	MPDIoU [7]	79.6	69.2	76.7	40.5	71.8	90.3	81.1	63.3
	CIoU	79.3	69.8	76.5	39.9	69.7	90.6	82.4	63.4
	DIoU	78.9	68.9	76.1	42.6	70.5	89.1	81.7	63.2
	EIoU	79.3	68.2	75.5	42.6	68.5	89.0	81.2	63.4
	SIoU	79.1	67.4	75.3	42.0	68.4	89.0	81.2	62.5
	WIoU	76.3	69.3	71.9	36.2	65.1	87.3	76.0	59.2
	Shape-IoU [65]	77.9	69.5	75.5	42.1	68.9	89.7	80.5	63.1

**Table 5:** Model ablation experiments on the RUOD dataset.

Model	Method	SPDConv	PSA	SCDown	MPDIoU	mAP_50_ (%)	mAP_50-95_ (%)	Parameters (M)	Model Size (MB)	GFLOPs	FPS
Baseline	a					86.6	63.2	3.00	6.2	8.1	260
	b	√				86.5	62.7	2.68	5.6	7.5	230
	c		√			87.5	64.0	3.12	6.5	8.1	226
	d			√		86.4	63.1	2.84	5.9	8.0	244
	e				√	87.4	64.2	3.00	6.2	8.1	262
	f	√	√			86.5	63.2	3.04	6.3	7.8	217
	g	√		√		86.0	62.3	2.62	5.5	7.5	208
	h	√			√	85.7	61.3	2.79	5.8	7.6	219
	i	√	√	√		87.0	63.6	2.87	6.0	7.7	197
	j	√	√		√	87.3	63.9	3.04	6.3	7.8	226
	k	√		√	√	87.0	63.8	2.62	5.5	7.5	247
	l		√	√		87.5	64.3	3.09	6.5	8.2	221
	m		√		√	87.9	65.0	3.25	6.8	8.3	242
	n		√	√	√	87.9	65.1	3.09	6.5	8.2	234
	o			√	√	87.1	63.9	2.84	5.9	8.0	275
	p [ours]	√	√	√	√	87.3	63.6	2.87	6.0	7.7	210

## Data Availability

Derived data supporting the findings of this study are available from the corresponding author upon request.
